# Liquid Biphasic Electric Partitioning System as a Novel Integration Process for Betacyanins Extraction From Red-Purple Pitaya and Antioxidant Properties Assessment

**DOI:** 10.3389/fchem.2019.00201

**Published:** 2019-04-03

**Authors:** Hui Yi Leong, Yu-Kaung Chang, Chien Wei Ooi, Chung Lim Law, Advina Lizah Julkifle, Pau Loke Show

**Affiliations:** ^1^Bioseparation Research Group, Department of Chemical and Environmental Engineering, Faculty of Science and Engineering, University of Nottingham Malaysia, Semenyih, Malaysia; ^2^Department of Chemical Engineering, Graduate School of Biochemical Engineering, Ming Chi University of Technology, Taipei, Taiwan; ^3^Chemical Engineering Discipline, School of Engineering, Monash University Malaysia, Bandar Sunway, Malaysia; ^4^Department of Chemical and Environmental Engineering, Faculty of Science and Engineering, University of Nottingham Malaysia, Semenyih, Malaysia; ^5^Crops For the Future, Semenyih, Malaysia

**Keywords:** antioxidant, betacyanins, electric system, integration process, liquid biphasic partitioning system, red-purple pitaya

## Abstract

Nowadays, downstream bioprocessing industries inclines towards the development of a green and high efficient bioseparation technology. Betacyanins are presently gaining higher interest in the food science as driven by their high tinctorial strength and health promoting functional properties. In this study, a novel green integration process of liquid biphasic electric partitioning system (LBEPS) was proposed for betacyanins extraction from peel and flesh of red-purple pitaya. Initially, the betacyanins extraction using LBEPS with initial settings was compared with that of liquid biphasic partitioning system (LBPS), and the results revealed that both systems demonstrated a comparable betacyanins extraction. This was followed by further optimizing the LBEPS for better betacyanins extraction. Several operating parameters including operation time, voltage applied, and position of graphitic electrodes in the system were investigated. Moreover, comparison between optimized LBEPS and LBPS with optimized conditions of electric system (as post-treatment) as well as color characterization and antioxidant properties assessment were conducted. Overall, the betacyanins extraction employing the optimized LBEPS showed the significant highest values of betacyanins concentration in alcohol-rich top phase (C_*t*_) and partition coefficient (K) of betacyanins from peel (99.256 ± 0.014% and 133.433 ± 2.566) and flesh (97.189 ± 0.172% and 34.665 ± 2.253) of red-purple pitaya. These results inferred that an optimal betacyanins extraction was successfully achieved by this approach. Also, the LBEPS with the peel and flesh showed phase volume ratio (V_*r*_) values of 1.667 and 2.167, respectively, and this indicated that they have a clear biphasic separation. In addition, the peel and flesh extract obtained from the optimized LBEPS demonstrated different variations of red color as well as their antioxidant properties were well-retained. This article introduces a new, reliable, and effective bioseparation approach for the extraction of biomolecules, which is definitely worth to explore further as a bioseparation tool in the downstream bioprocessing.

## Introduction

Lately, betacyanins are of growing interest in the applications of food processing, such as foods, nutraceuticals, and pharmaceuticals, owing to their versatile properties including attractive visual attributes, pigments stability between pH 3 and 7, natural coloring feature (E-162), powerful antioxidant and health promoting functional properties (Ciriminna et al., 2018; Leong et al., 2018c). The most common and simplest structure of betacyanin found in plants is betanin, also known as betanidin-5-O-β-glucoside. Betacyanins are red-violet pigments which is an important constituent of betalains. Betalains are water-soluble nitrogen-containing natural pigments that have presently received attention as a source of natural colorant (Aberoumand, 2011; Carocho et al., 2015). The pigments contain a chromophore of betalamic acid, in which its conjugation with *cyclo*-3,4-dihydroxyphenylalanine can produce the red-violet betacyanins, whereas yellow-orange betaxanthins can be synthesized through the conjugation of betalamic acid with different amino acids or amines. As compared to betaxanthins, betacyanins are known to be more stable in terms of their structural aspects (Azeredo, 2009). One of the rich sources of betacyanins is red-purple pitaya (*Hylocereus polyrhizus*), in addition to red beetroot and other Caryophyllales. Red-purple pitaya is a type of *Hylocereus* species which belongs to the family of Cactaceae. It is a red-skinned fruit with red-purple flesh and black seeds. Additionally, red-purple pitaya is high in nutritional contents and is a promising source of betacyanins, and hence, it possesses positive effects on health (Stintzing and Carle, 2007; Moreno et al., 2008; Dembitsky et al., 2011; Esatbeyoglu et al., 2015; Khan and Giridhar, 2015; Ciriminna et al., 2018).

Extraction of betalains from various plant sources normally utilizes conventional solid-liquid extraction approaches, such as maceration and Soxhlet extraction (Castellar et al., 2003; Chong et al., 2014; Ramli et al., 2014; Celli and Brooks, 2017). These extraction procedures are reported to have limitations, for example, inefficiency, time-, energy-, and cost-consuming, lower yields production as well as not eco-friendly (Wang and Weller, 2006; Dai and Mumper, 2010; Azmir et al., 2013; Ciriminna et al., 2018). Development of a green, reliable, economically effective, and high efficient bioseparation technology is now a rapidly growing field in biotechnology industries including downstream bioprocessing industries (Tang and Zhao, 2009; Chemat et al., 2017; Sankaran et al., 2018). To address this need, non-conventional innovative extraction techniques for the betalains extraction have been recently developed. Indeed, several green extraction techniques, such as ultrasound (Ramli et al., 2014; Laqui-Vilca et al., 2018), microwave (Bastos and Gonçalves, 2017), pulsed electric field (Fincan et al., 2004), and high pressure CO_2_ (Ciriminna et al., 2018) have been applied along with conventional extraction methods for the betalains extraction, and a better extraction efficiency of betalains at a reduced extraction time were reported (Celli and Brooks, 2017; Xu et al., 2017). Other than that, application of a liquid biphasic system, such as aqueous two-phase system (ATPS) (Chethana et al., 2007; Chandrasekhar et al., 2015; Santos et al., 2018) and liquid biphasic flotation (LBF) system (Leong et al., 2018a) for the separation, purification, and concentration of betalains from plants have been studied. The liquid biphasic system is well-known as an easy, scalable, time-, cost-, and energy-saving, effective as well as mild and green separation approach for many biotechnological products (Show et al., 2013; Yau et al., 2015)(Zimmermann et al., 2017).

Taking the above into account, in the present study, an integration process of liquid biphasic partitioning and electric system, namely liquid biphasic electric partitioning system (LBEPS) was proposed for betacyanins extraction from red-purple pitaya. In this study, both peel and flesh of red-purple pitaya were fully utilized for their betacyanins extractions. Liquid biphasic partitioning system (LBPS) is a new and green liquid biphasic system for separation of biomolecules as well as possesses the gifted advantages of the current liquid biphasic system. The LBPS was integrated with electricity treatment (electric system) in this study as to further enhance the LBPS for improving biomolecules separation. Electricity treatment is a non-thermal processing approach that ameliorates extraction efficiency of biomolecules. The betacyanins extraction using LBEPS was first compared with that of LBPS, and then followed by optimization study on the LBEPS for the betacyanins extraction. In addition, comparison between optimized LBEPS and LBPS with optimized conditions of electric system (as post-treatment) as well as color characterization and antioxidant properties assessment were carried out. To the best of our knowledge, this is the first article reporting the betacyanins extraction process by employing LBEPS. It is worth noting that the LBEPS is a novel green integration process, and it is also of significance that the separation of biomolecules was performed for the first time using LBEPS.

## Materials and Methods

### Materials

Red-purple pitaya was purchased from a local fruit stall in Semenyih, Selangor Darul Ehsan, Malaysia. Ultrapure water produced from Milli-Q integral water purification system (Merck, Darmstadt, Germany) was used throughout this experiment. Graphitic electrodes which were modified from 2B pencil leads (diameter: 2 mm) were acquired from My Family Art & Stationery (Semenyih, Selangor Darul Ehsan, Malaysia). Absolute ethanol, dipotassium hydrogen phosphate (K_2_HPO_4_), sodium chloride (NaCl), sodium bicarbonate (NaHCO_3_), iron (III) chloride hexahydrate (FeCl_3_·6H_2_O), and iron (II) sulfate heptahydrate (FeSO_4_·7H_2_O) were purchased from R&M Chemicals (Selangor Darul Ehsan, Malaysia). Acetic acid (CH_3_COOH) and sodium acetate trihydrate (C_2_H_3_NaO_2_·3H_2_O) were obtained from Merck (Darmstadt, Germany). Hydrochloric acid (HCl) was purchased from Fisher Scientific (Selangor Darul Ehsan, Malaysia). 2,4,6-tripyridyl-s-triazine (TPTZ), 2,2′-azino-bis(3-ethylbenzothiazoline-6-sulfonic acid) diammonium salt (ABTS), potassium persulfate (K_2_O_8_S_2_), 6-hydroxy-2,5,7,8-tetramethychroman-2-carboxylic acid (Trolox), Folin-Ciocalteu (F-C) reagent and gallic acid were acquired from Sigma-Aldrich (St. Louis, MO, USA). All the above mentioned chemicals were of analytical grade (purity > 95%).

### Apparatus

A regulated dual direct current (DC) power supply (ATTEN APS3003S-3D, 30 V/3 A*^2^) (Mobicon-Remote Electronic, Petaling Jaya, Selangor Darul Ehsan, Malaysia) was used to supply electricity in this experiment (i.e., electric system), and was kindly provided by the Department of Electrical and Electronic Engineering, University of Nottingham Malaysia.

### Preparation of Crude Extract

The preparation of crude extract was conducted in dim light condition in order to minimize its pigment losses. The peel and flesh of red-purple pitaya were firstly cut into thin pieces after washed and dried with tissue towel, subsequently, they were stored at −80°C for 48 h. To prepare dried crude extract (DE), the sample was freeze dried at −30°C and 0.37 atm for 48 h using a freeze dryer (CHRIST Alpha 1-2 LDplus, Germany). After that, the freeze-dried crude extract was ground into powder using a grinder (Tefal Blendforce, Triple'Ax Technology 400 Watt, Malaysia). The DE of the peel and flesh of red-purple pitaya were stored at −20°C for further use.

### Betacyanins Extraction Using LBEPS

**Figure 1:**
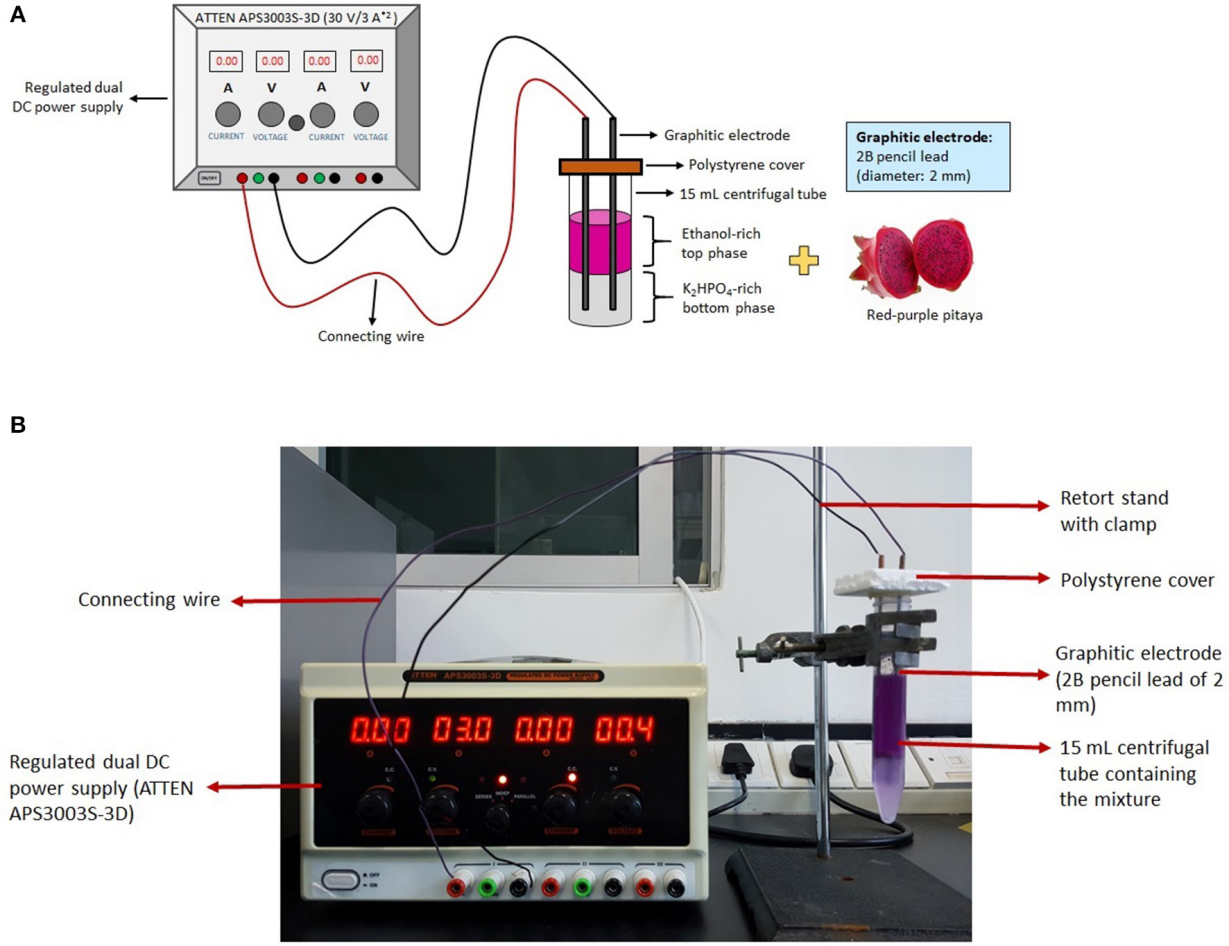
**(A)** Schematic diagram of LBEPS for betacyanins extraction from peel and flesh of red-purple pitaya. **(B)** Experimental setup for the betacyanins extraction using LBEPS.

The LBEPS was created by equipping two graphitic electrodes into the LBPS. The electrodes (anode and cathode; former to apply voltage and latter to detect current flow) were connected to a regulated dual DC power supply in order to supply electricity continuously (Figure 1). A 10 g LBPS with optimized conditions (Leong et al., 2018b) was further incorporated with electric system (i.e., LBEPS). The operating parameters of the 10 g LBEPS including operation time, voltage apply, and position of graphitic electrodes were optimized using one-factor-at-a-time (OFAT) approach for the betacyanins extraction from peel and flesh of red-purple pitaya. The initial settings of the LBEPS were 15 min of operation time, 3 V of voltage applied and graphitic electrodes positioned at bottom phase (Table 1). The experiment was conducted at room temperature (25 ± 1°C).

**Table 1:** Operating parameters of LBEPS for betacyanins extraction from peel and flesh of red-purple pitaya.

**No**.	**Condition**	**Initial setting**	**Variables**	**Unit**
1.	10 g LBPS with optimized conditions (constant throughout the experiment) (%, w/w) (Leong et al., 2018b)			
	Peel		Flesh	
	1% of DE		1% of DE	
	27% of undiluted ethanol		33% of undiluted ethanol	
	20% of K_2_HPO_4_ solution		20% of K_2_HPO_4_ solution	
	50% of purified water		44% of purified water	
	2% of 0.4M NaCl		2% of 0.2M NaCl	
**ELECTRIC SYSTEM**				
2.	Operation time	20	10, 15, 25, 30	min
3.	Voltage apply	3	1, 2, 4, 5	V
4.	Position of graphitic electrodes	Bottom phase	Top and middle phase; middle position refers to the interphase between top and bottom phase	N/A

#### Comparison Between LBPS and LBEPS

A 10 g LBPS was prepared in a 15 mL graduated centrifugal tube by mixing phase-forming components and DE in accordance with their respective compositions (%, w/w), as shown in Table 1. After thorough mixing of all the components by gentle agitation, the mixture was centrifuged at 3,000 rpm for 20 min to induce a phase separation. On the other hand, in the 10 g LBEPS, a biphasic system was firstly formed; the phase-forming components were mixed well and centrifuged at 3,000 rpm for 5 min, subsequently, 1% of DE was added to the biphasic system and gently mixed. After that, the mixture was supplied with electricity, as mentioned in section Betacyanins Extraction using LBEPS (i.e., LBEPS with initial settings). The Volumes of the top and bottom phase in LBPS and LBEPS were then measured, followed by the collection of sample from both phases for analysis of total betacyanins content (TBC).

### Comparison Between Optimized LBEPS and LBPS With Optimized Conditions of Electric System as Post-treatment

A 10 g LBPS was firstly prepared, as mentioned in section Comparison Between LBPS and LBEPS, subsequently, the optimized conditions of the electric system (operation time, voltage applied, and position of graphitic electrodes in the LBEPS) were used in further treatment of the mixture (i.e., post-treatment). The results obtained were compared with optimized LBEPS.

### Analytical Procedures

#### Color Characterization

Lightness (L^*^), redness (a^*^), and yellowness (b^*^) of the peel and flesh extract of red-purple pitaya were analyzed using a colorimeter (Lovibond LC 100, model RM 200; The Tintometer Ltd., United Kingdom). Additionally, their hue angle (h°) and chroma (C^*^) were calculated according to Equations (1, 2), respectively.


(1) $$h^{{}^{\circ}}=\tan^{-1}\frac{b^{*}}{a^{*}}$$

(2) $$C^{*}=\sqrt{a^{*2}+b^{*2}}$$

#### Analysis of Total Betacyanins Content (TBC)

The TBC in the crude extract was analyzed using a UV-vis spectrophotometer (UV-1,800; Shimadzu Corporation, Japan) at 538 nm. The TBC was expressed as mg of betanin equivalents (BEs) per 100 g of crude extract, and was calculated according to Equation (3) (Ramli et al., 2014; Leong et al., 2018a,b):


(3) $$\text{TBC}=\;\frac{{\text{A}}_{\text{538}}\;\times \text{ MW }\times \text{ V }\times \text{ DF }}{\varepsilon \;\times \text{ L }\times \text{ W}}\;\times \text{100}$$

Where A_538_ = absorbance value at 538 nm, MW = molecular weight of betanin (550 g.mol^−1^), V = volume of sample (mL), DF = dilution factor, ε = molar extinction coefficient of betanin (65,000 L.mol^−1^.cm^−1^), L = path length of cuvette (1 cm), W = weight of crude extract (g).

#### Analysis of Total Phenolic Content (TPC)

The Folin-Ciocalteu (F-C) method as described in Singleton et al. (1999), Fu et al. (2011), and Hajimahmoodi et al. (2013) was employed to analyze the TPC in the crude extract. A diluted F-C reagent which consists of 10 mL of F-C reagent and 90 mL of purified water was freshly prepared. Next, 100 μL of sample or gallic acid solution (i.e., standard) was mixed with 500 μL of diluted F-C reagent. The mixture was then incubated for 5 min at room temperature under dark condition. Subsequently, 2 mL of 60 g/L of NaHCO_3_ solution was added to the mixture. The mixture was mixed well and kept for 90 min at room temperature under dark condition. The absorbance value of the mixture was measured at 725 nm using a UV-vis spectrophotometer. The TPC was expressed as mg of gallic acid equivalents (GAEs) per 100 g of crude extract.

#### Analysis of Ferric Reducing Antioxidant Power (FRAP)

The FRAP in the crude extract was analyzed using method as described in literature (Benzie and Strain, 1996; Fu et al., 2011; Leong et al., 2018a). The FRAP reagent was freshly prepared; 10 mL of 10 mmol/L TPTZ solution (0.0031 g of TPTZ in 1 mL of 40 mmol/L HCl) and 10 mL of 20 mmol/L FeCl_3_·6H_2_O solution (0.0054 g/mL) for every 100 mL of 300 mmol/L acetate buffer (pH 3.6; mixture of 3.1 g of C_2_H_3_NaO_2_·3H_2_O with 16 mL of CH_3_COOH per liter of purified water). The FRAP reagent was preheated to 37°C before use. The FRAP assessment was carried out by mixing 100 μL of sample or FeSO_4_·7H_2_O solution (i.e., standard), 300 μL of purified water and 3 mL of FRAP reagent. The mixture was subsequently incubated for 4 min at 37°C. The absorbance value of mixture was measured at 593 nm using a UV-vis spectrophotometer. The result was expressed as μmol of Fe(II) per g of crude extract.

#### Analysis of Trolox Equivalent Antioxidant Capacity (TEAC)

The ABTS radical (ABTS**·**) method as described in Re et al. (1999), Fu et al. (2011) was employed to analyze the TEAC in the crude extract. The ABTS**·** stock solution (mixture of 7 mmol/L of ABTS solution and 2.45 mmol/L of K_2_O_8_S_2_ solution at v:v ratio of 1:1) was first prepared, and was then incubated for 12–16 h at room temperature under dark condition. Next, the ABTS**·** stock solution was diluted with ethanol to reach an absorbance value of 0.70 ± 0.05 at 734 nm, and was incubated at 30°C. As to analyze the TEAC, 100 μL of sample or Trolox solution (i.e., standard) or ethanol (i.e., control) was mixed with 3.8 mL of diluted ABTS**·** solution. The absorbance value of the mixture was measured at 734 nm using a UV-vis spectrophotometer after 6 min of incubation at 30°C. The result was expressed as μmol of Trolox equivalents (TEs) per g of crude extract. The percentage of scavenging on ABTS**·** was calculated using Equation (4) (Leong et al., 2018a):


(4) $$\text{Percentage of scavenging }(\%)=\;\frac{\text{control - sample or standard}}{\text{control}}\times \text{100}$$

### Calculations

Partition coefficient (K) of betacyanins in the LBPS and LBEPS was calculated according to Equation (5) (Leong et al., 2018a,b):


(5) $$\text{K}=\;\frac{{\text{TBC}}_{\text{t}}}{{\text{TBC}}_{\text{b}}}$$

Where TBC_t_ and TBC_b_ are TBC in the alcohol-rich top phase and salt-rich bottom phase at equilibrium, respectively.

Betacyanins concentration (%) in alcohol-rich top phase (C_*t*_) and salt-rich bottom phase (C_*b*_) was calculated according to Equations (6, 7), respectively:


(6) $${\text{C}}_{\text{t}}(\%)=\frac{\text{TBC in top phase }}{\text{TBC in crude extract }}\;\times \text{100 }=\;\frac{{\text{TBC}}_{\text{t}}}{{\text{TBC}}_{\text{t}}\text{+ }{\text{TBC}}_{\text{b}}}\;\times \text{100}$$

(7) $${\text{C}}_{\text{b}}(\%)=\frac{\text{TBC in bottom phase }}{\text{TBC in crude extract }}\;\times \text{100 }=\;\frac{{\text{TBC}}_{\text{b}}}{{\text{TBC}}_{\text{t}}\text{+ }{\text{TBC}}_{\text{b}}}\;\times \text{100}$$

Phase volume ratio (V_*r*_) is defined as ratio of the volume of alcohol-rich top phase to the volume of salt-rich bottom phase at equilibrium, and was calculated according to Equation (8).


(8) $${\text{V}}_{\text{r}}=\;\frac{{\text{V}}_{\text{t}}}{{\text{V}}_{\text{b}}}$$

Where V_*t*_ and V_*b*_ are volume of the alcohol-rich top phase and salt-rich bottom phase at equilibrium, respectively.

### Statistical Analysis

The statistical analysis was performed using IBM SPSS statistics software (SPSS version 23.0 for window, IBM Corporation, Armonk, New York, United States). Triplicate readings were recorded and were used in the analysis, and the values were expressed as mean ± standard deviation (SD). The data were subjected to One-Way analysis of variance, and the mean differences were compared using Tukey HSD *post-hoc* multiple comparisons test. The data were considered for their statistically significant difference where *p* < 0.05. Moreover, the relationship among the antioxidant properties was analyzed using Pearson's correlation test.

## Results and Discussion

### Comparison Study of Betacyanins Extraction Using LBPS and LBEPS

**Figure 2:**
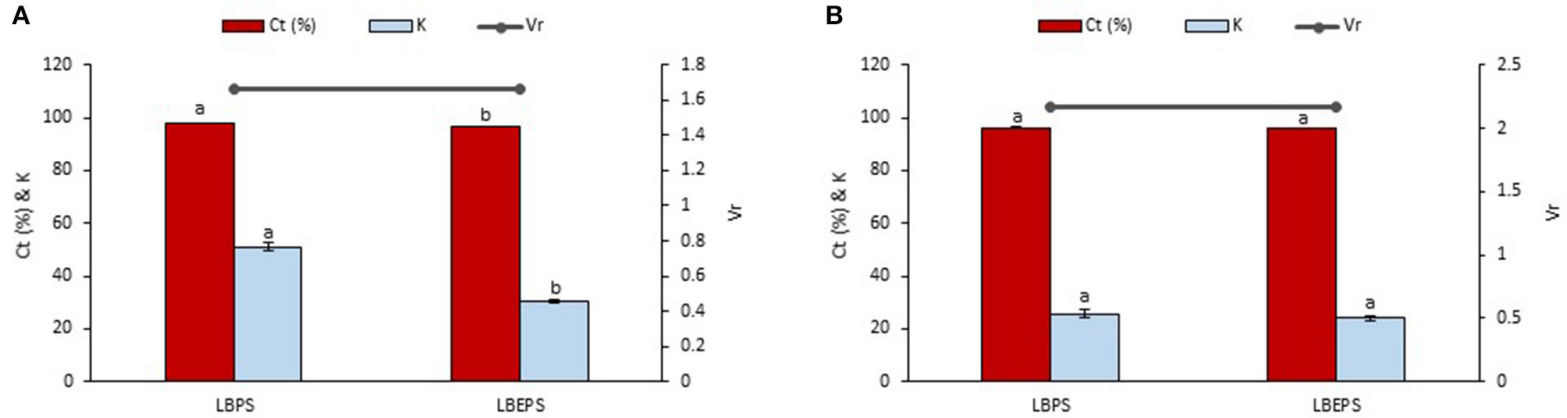
Comparison study of betacyanins extraction from **(A)** peel and **(B)** flesh of red-purple pitaya using LBPS and LBEPS with initial settings. Values are mean ± SD of triplicate readings. Different letter(s) represent a significant difference (*p* < 0.05) using Tukey's test within C_*t*_ and K.

An initial study was conducted by comparing the betacyanins extraction from peel and flesh of red-purple pitaya using LBPS with optimized conditions (Leong et al., 2018b) and LBEPS (i.e., an integration process of LBPS and electricity treatment) with initial settings. Our findings showed that the betacyanins extraction from the peel using LBPS was slightly better than that of using LBEPS. The application of LBPS showed the significant highest values of C_*t*_ (98.080 ± 0.051%) and K of betacyanins (51.097 ± 1.354) compared to that of the values obtained from LBEPS (C_*t*_: 96.820 ± 0.046% and K of betacyanins: 30.450 ± 0.459). Both systems showed a similar value of V_*r*_ (1.667) (Figure 2A). Likewise, a similar trend was observed for the betacyanins extraction from the flesh, as shown in Figure 2B. However, the betacyanins extraction from the flesh using LBPS showed the non-significant highest values of C_*t*_ (96.256 ± 0.207%) and K of betacyanins (25.764 ± 1.525) as compared to that of the LBEPS (C_*t*_: 96.010 ± 0.144% and K of betacyanins: 24.086 ± 0.911) (*p* > 0.05). Both systems also showed a similar value of V_*r*_ (2.167). The similar value of V_*r*_ revealed that the biphasic separation was not affected by the difference in these two extraction methods. In LBPS, centrifugation process alongside biphasic system was utilized in the betacyanins extraction, whereas electricity treatment alongside biphasic system was applied to the betacyanins extraction in LBEPS. Both approaches are different in terms of their working mechanisms. Although LBPS resulted in the slightly higher betacyanins extraction, we decided to further optimize our LBEPS for the betacyanins extraction in this study. The reasons are that both systems showed a comparable betacyanins extraction from red-purple pitaya and LBEPS seems to be promising for achieving a more efficient betacyanins extraction than LBPS due to the fact that its conditions used in this study could still be optimized.

### Effect of the Operation Time in LBEPS

**Figure 3:**
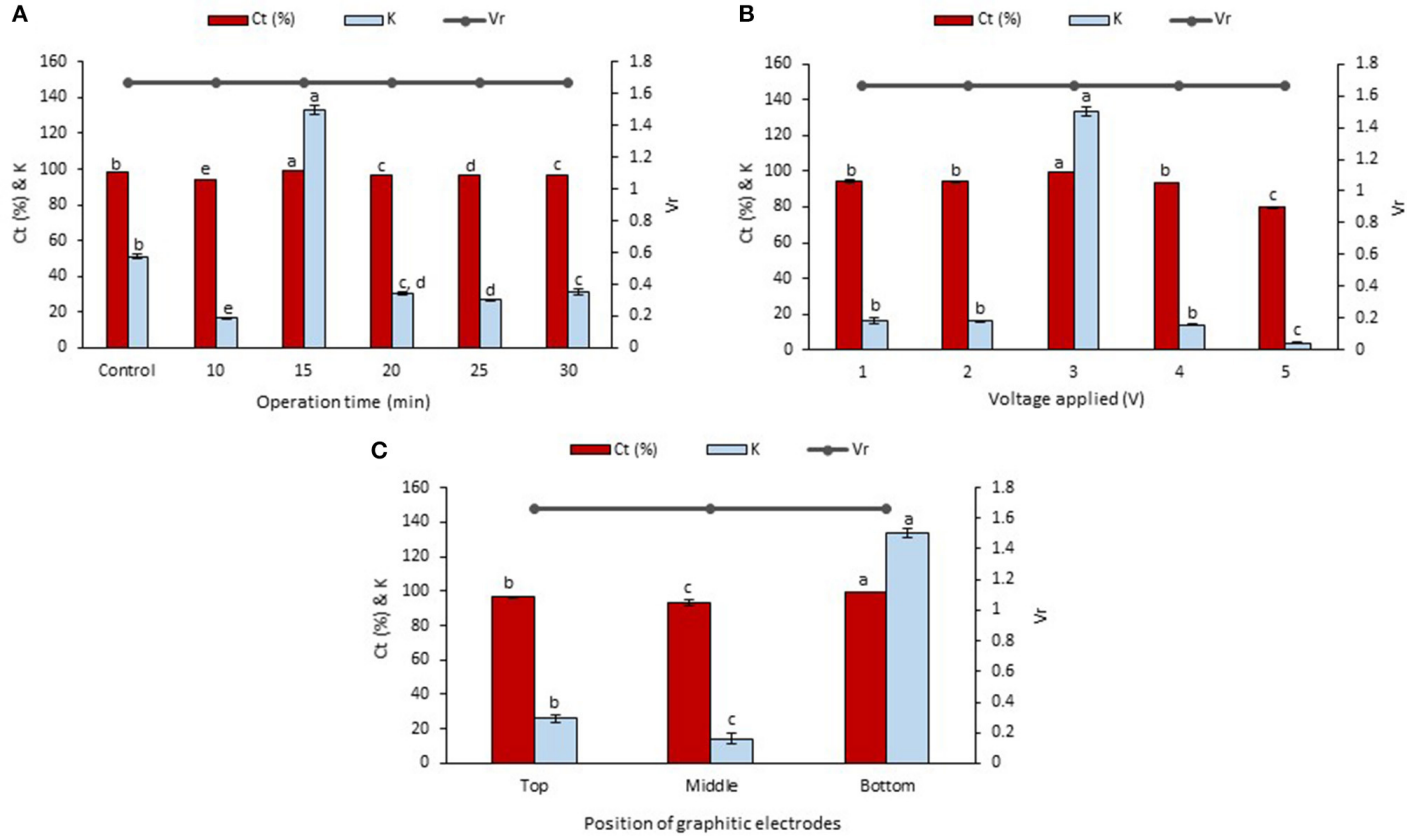
Effects of various parameters in LBEPS for betacyanins extraction from peel of red-purple pitaya; OFAT approach was applied in the optimization study: **(A)** operation time (control: LBPS), **(B)** voltage applied and **(C)** position of graphitic electrodes. Values are mean ± SD of triplicate readings. Different letter(s) represent a significant difference (*p* < 0.05) using Tukey's test within C_*t*_ and K.

**Figure 4:**
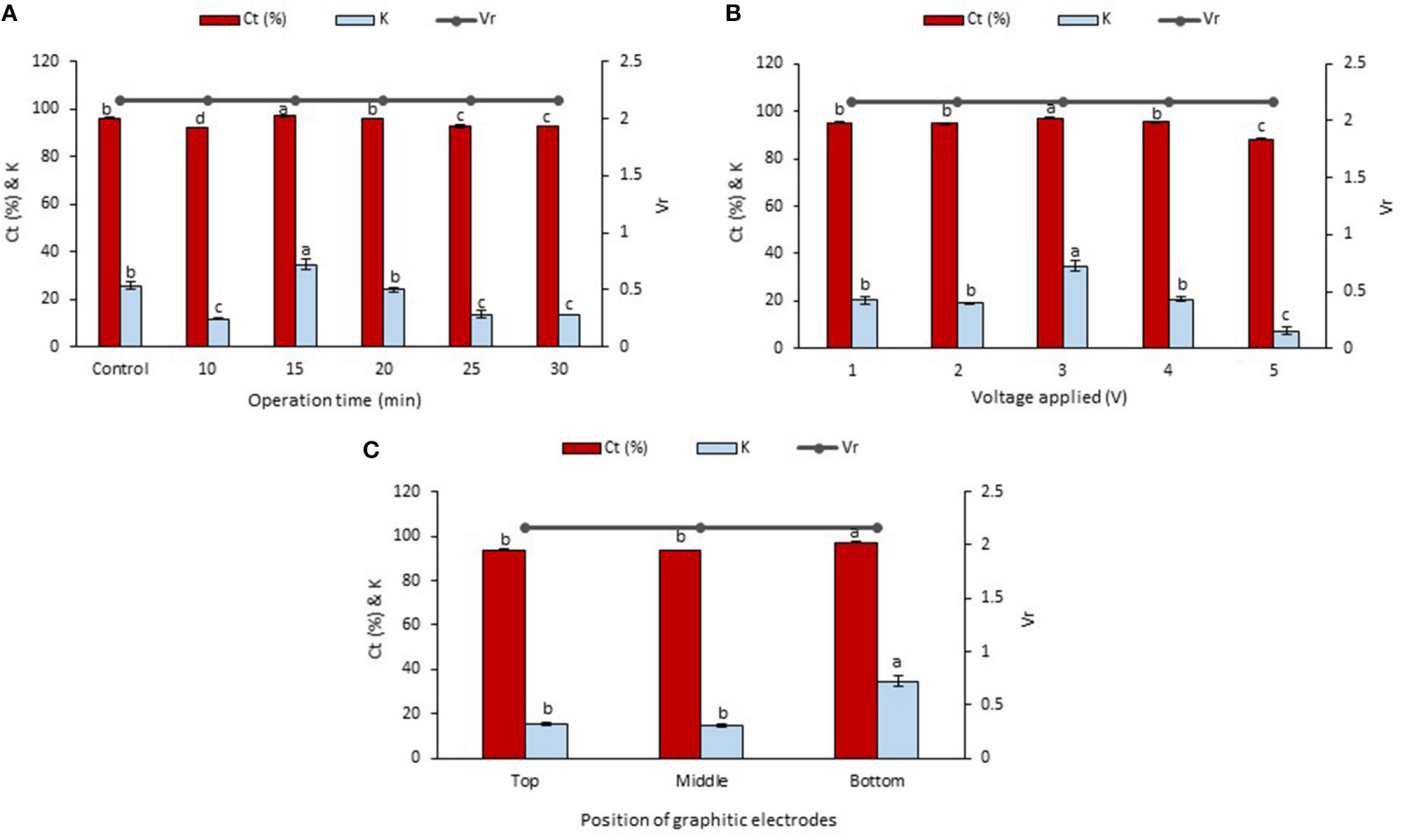
Effects of various parameters in LBEPS for betacyanins extraction from flesh of red-purple pitaya; OFAT approach was applied in the optimization study: **(A)** operation time (control: LBPS), **(B)** voltage applied and **(C)** position of graphitic electrodes. Values are mean ± SD of triplicate readings. Different letter(s) represent a significant difference (*p* < 0.05) using Tukey's test within C_*t*_ and K.

The betacyanins extraction from the peel and flesh using LBEPS were further studied by optimizing the operation time (10–30 min) in the system. Other parameters of the LBEPS included 3 V of voltage applied and graphitic electrodes located at bottom phase in the system. As depicted in Figure 3A, an increase in the operation time from 10 to 15 min in the LBEPS greatly improved the betacyanins extraction from the peel, and further extending the operation time up to 30 min resulted in a slightly lower betacyanins extraction compared to that of with 15 min of operation time. Meanwhile, LBEPS with 15 min of operation time showed a better betacyanins extraction compared to LBPS with 20 min of operation time (control). The significant highest values of C_*t*_ and K of betacyanins obtained from the LBEPS with 15 min of operation time were 99.256 ± 0.014% and 133.433 ± 2.566, respectively, among the others. The control and LBEPS with different operation times showed a similar V_*r*_ value of 1.667, and this inferred that their biphasic separation was not affected by the different extraction methods (as mentioned earlier in section Comparison Study of Betacyanins Extraction Using LBPS and LBEPS). Also, a similar trend was noted for the betacyanins extraction from the flesh, as shown in Figure 4A. The significant highest values of C_*t*_ and K of betacyanins obtained from the LBEPS with 15 min of operation time were 97.189 ± 0.172% and 34.665 ± 2.253, respectively, among the others. The control and LBEPS with different operation times also showed a similar V_*r*_ value (2.167).

Collectively, a higher betacyanins extraction from the peel and flesh was achieved through the use of LBEPS with 15 min of operation time compared to that of with LBPS with 20 min of operation time as well as to that of with LBEPS with other operation times. Application of LBEPS can shorten the extraction time from 20 to 15 min; compared to control (i.e., LBPS). The possible reason might be due to the different working extraction mechanisms between LBPS and LBEPS, as mentioned earlier in section Comparison Study of Betacyanins Extraction Using LBPS and LBEPS. The LBEPS uses electricity treatment alongside biphasic system for the betacyanins extraction, while LBPS employs centrifugation process alongside biphasic system for the betacyanins extraction. The electricity treatment occurred between the two graphitic electrodes is suggested to enhance the betacyanins extraction and cause a higher extraction efficiency compared to the centrifugation process. Other than that, as compared among the 10–30 min of operation time of LBEPS, 15 min of the operation time was noted to be the most effective extraction time. This could be explained by the mass transfer energy of the system reaching equilibrium and the maximum at 15 min. Moreover, a longer extraction time can cause oxidation of betacyanins due to their highly sensitive features (Esatbeyoglu et al., 2015; Khan, 2016; Celli and Brooks, 2017; Ciriminna et al., 2018; Leong et al., 2018a). Hence, 15 min of operation time was chosen for the betacyanins extraction using LBEPS.

### Effect of the Voltage Applied in LBEPS

**Figure 5:**
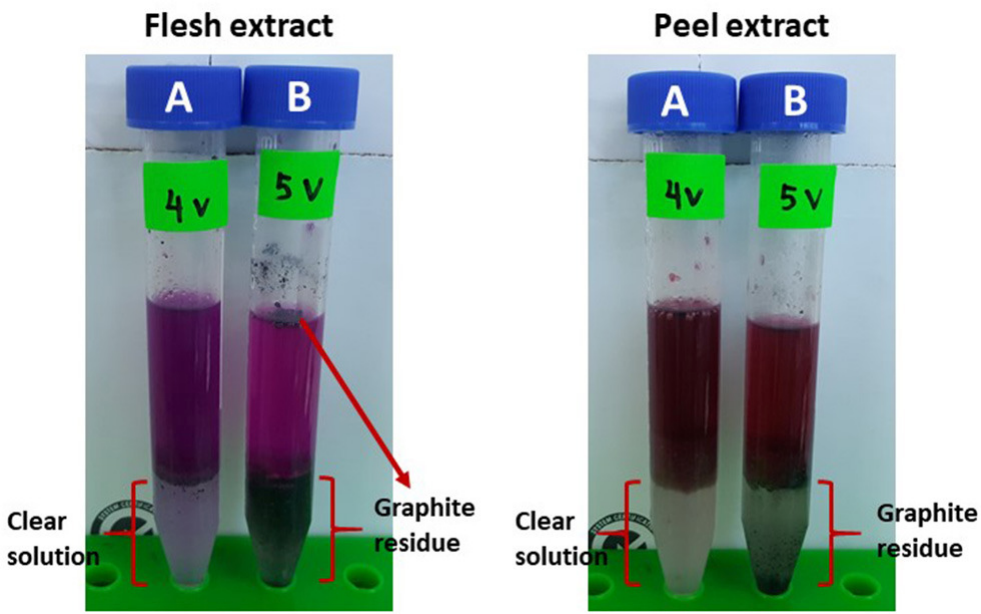
Effect of different voltages applied in LBEPS; comparison between **(A)** 4 V and **(B)** 5 V of voltage applied for the peel and flesh extract.

Effect of the voltages applied ranging from 1 to 5 V in the LBEPS for betacyanins extraction from peel and flesh of red-purple pitaya was investigated, and the results are presented in Figures 3B, 4B, respectively. The betacyanins extraction using LBEPS with 3 V of voltage applied showed the significant highest values of C_*t*_ and K of betacyanins from the peel (99.256 ± 0.014% and 133.433 ± 2.566, respectively) and flesh (97.189 ± 0.172% and 34.665 ± 2.253, respectively). Meanwhile, the values of C_*t*_ and K of betacyanins from the peel and flesh obtained from the betacyanins extraction using LBEPS with 1, 2, and 4 V of voltage applied were noted to be no significant difference among them (*p* > 0.05). However, the betacyanins extraction using LBEPS with 5 V of voltage applied showed the significant lowest values of C_*t*_ and K of betacyanins from the peel (79.737 ± 0.419% and 3.937 ± 0.101, respectively) and flesh (88.231 ± 0.162% and 7.498 ± 0.116, respectively). Additionally, with the 5 V of voltage applied, the graphitic electrodes were oxidized and graphite residue was observed in the salt-rich bottom phase, as shown in Figure 5. Furthermore, V_*r*_ values in the LBEPS with different voltages applied for the peel and flesh showed that different voltages do not influence the biphasic separation much, due to their similar values among the others; LBEPS with peel: 1.667 and LBEPS with flesh: 2.167.

Our results inferred that LBEPS with 3 V of voltage applied achieved an optimal betacyanins extraction from the peel and flesh as shown by their significant highest values of C_*t*_ and K of betacyanins. The electricity treatment supplied by the 3 V of voltage applied in the system is proved to have the highest electropermeabilization of red-purple pitaya membrane structure, in which the cell membrane structure was disrupted by short and intense electric pulses, and the increased cell membrane permeability led to the release of more betacyanins from red-purple pitaya to the extractive solvent in the system. This eventually increase the extraction efficiency of betacyanins. The betacyanins adhered to the electrodes and moved along from bottom to top phase of the system via electricity treatment. This electricity treatment is known as a non-thermal process where an external electricity is supplied to a substrate. Additionally, the effectiveness of the electricity treatment strongly depends on the electrical field strength, specific energy input, treatment duration, substance to be treated etc. (Azmir et al., 2013; Boussetta and Vorobiev, 2014; Celli and Brooks, 2017; Chemat et al., 2017; Xu et al., 2017). A study conducted by Roselló-Soto et al. (2015) reported that high voltage electrical discharges was the most effective pretreatment for the extraction of olive kernels among the other pretreatments of pulsed electric field and ultrasound, due to its highest extraction efficiency. This result was explained by the occurrence of propagation of the shock waves and explosion of cavitation bubbles during the pretreatment of olive kernel induced by the application of electrical discharges.

Moreover, in the present study, oxidation of the graphitic electrodes was observed when 5 V of voltage was applied in the LBEPS. In the same instance, this system showed the lowest betacyanins extraction from the peel and flesh. This could be explained by the fact that 10 g LBEPS cannot withstand the strong electricity treatment induced by the 5 V of voltage applied in the system. Also, the lowest betacyanins extraction might be caused by the presence of graphite residue due to oxidation of the graphitic electrodes, in which they degrade the betacyanins since betacyanins are highly sensitive pigments.

### Effect of the Position of Graphitic Electrodes in LBEPS

Subsequently, the LBEPS with 15 min of operation time and 3 V of voltage applied for the betacyanins extraction was further assessed using different positions of graphitic electrodes in the system; the electrodes were positioned at top, middle and bottom phase in the LBEPS. Our results revealed that the electrodes located at the bottom phase in LBEPS augmented the betacyanins extraction, with the significant highest values of C_*t*_ and K of betacyanins from the peel (99.256 ± 0.014% and 133.433 ± 2.566, respectively; Figure 3C) and flesh (97.189 ± 0.172% and 34.665 ± 2.253, respectively; Figure 4C). The top and middle position of the graphitic electrodes seemed to provide a slightly lower betacyanins extraction, owing to their smaller values of C_*t*_ and K of betacyanins. The possible explanation could be, in the bottom position, the electrodes have the maximum electrical field influence that allows betacyanins to move along from bottom to top phase. V_*r*_ values for the LBEPS in this case with the peel and flesh inferred that different positions of electrodes in the system do not have a significant impact on the biphasic separation, due to their similar values among the others; LBEPS with peel: 1.667 and LBEPS with flesh: 2.167.

### Comparison Study of Betacyanins Extraction Using Optimized LBEPS and LBPS With Optimized Conditions of Electric System as Post-treatment

**Figure 6:**
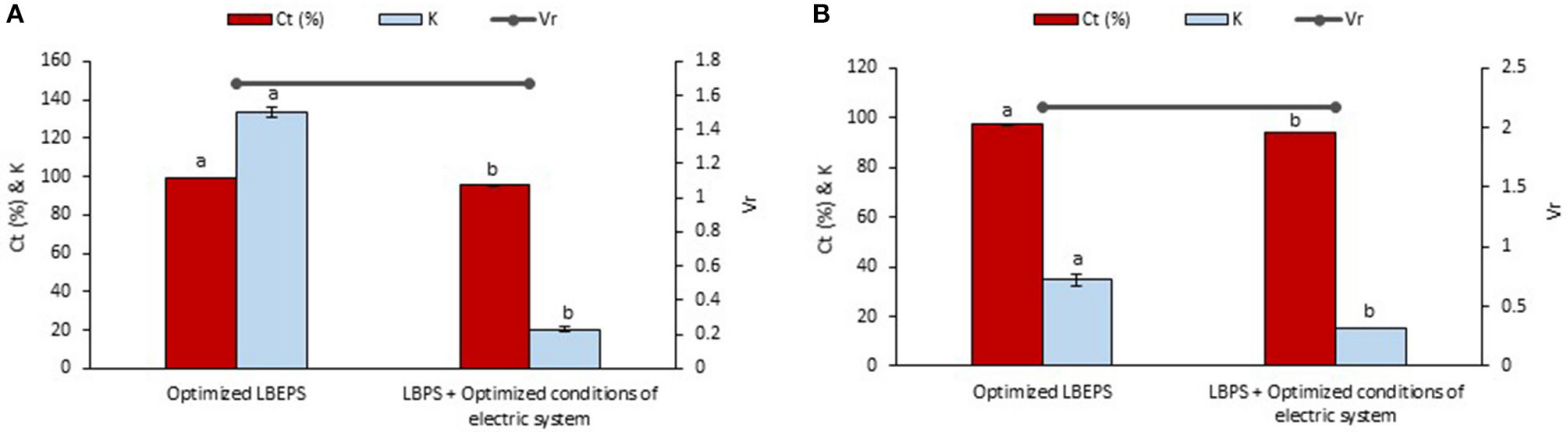
Comparison study of betacyanins extraction from **(A)** peel and **(B)** flesh of red-purple pitaya using the optimized LBEPS and LBPS with the optimized conditions of electric system (as post-treatment). Values are mean ± SD of triplicate readings. Different letter(s) represent a significant difference (*p* < 0.05) using Tukey's test within C_*t*_ and K.

Optimized conditions of electric system in the LBEPS included 15 min of operation time, 3 V of voltage applied and the electrodes located at bottom phase. The optimized LBEPS augmented the betacyanins extraction from peel and flesh of red-purple pitaya. As a result, these optimized conditions were incorporated to the LBPS as post-treatment for the betacyanins extraction, and the results obtained were compared with that of the optimized LBEPS (Figure 6). Our findings showed that the optimized LBEPS owned a better betacyanins extraction from the peel and flesh compared to that of using LBPS with the electric system as post-treatment (C_*t*_ and K of betacyanins from the peel were 95.296 ± 0.309% and 20.323 ± 1.431, respectively, as well as from the flesh were 93.825 ± 0.012% and 15.195 ± 0.032, respectively). The reason for the results obtained could be that the LBPS with electric system as post-treatment requires longer extraction time (i.e., 35 min) that might cause oxidation of betacyanins to occur. This eventually might reduce the betacyanins extraction. On the other hand, the V_*r*_ values are similar for both approaches. Therefore, an optimized LBEPS with 15 min of operation time, 3 V of voltage applied and the electrodes located at bottom phase was chosen for the betacyanins extraction from the peel and flesh of red-purple pitaya.

### Color Characterization

After the most effective extraction approach for the betacyanins extraction was determined, the color characterization of the extract was analyzed further. In this case, peel and flesh extract of red-purple pitaya (ethanol-rich top phase) used in the analysis were obtained from the optimized LBEPS. Both extracts are presented in different hues, lightness, saturations and intensities of color. They showed different degrees of lightness (L^*^), redness (a^*^), yellowness (b^*^), chroma (C^*^), and hue angle (h°), as presented in Table 2. The peel extract showed a higher degree of a^*^ and b^*^ as well as a smaller degree of L^*^, C^*^ and h° compared to that of the flesh extract. Our results revealed that the peel and flesh extract showed different ranges of red color. The peel extract showed a red-yellowish color (positive a^*^ and b^*^), whereas the flesh extract showed a red-bluish/purplish color (positive a^*^ and negative b^*^). The C^*^ and h° measure the basic tint and saturation of a color, respectively (Lancaster and Lister, 1997). Owing to their natural coloring attribute, the peel and flesh extract can be applied as natural colorants with different variations of red color.

**Table 2:** Color characterization of peel and flesh extract of red-purple pitaya obtained from the optimized LBEPS.

**Color parameter**	**Peel extract**	**Flesh extract**
L*	7.933 ± 0.306	11.033 ± 0.208
a*	23.633 ± 0.651	36.100 ± 0.529
b*	4.567 ± 0.306	−17.167 ± 0.153
C*	24.067 ± 0.651	39.967 ± 0.569
h°	10.933 ± 0.666	334.533 ± 0.153

L^*^, a^*^, b^*^, C^*^, and h° represent lightness, redness, yellowness, chroma, and hue angle, respectively. Values are mean ± SD of triplicate readings.

### Antioxidant Properties Assessment

**Figure 7:**
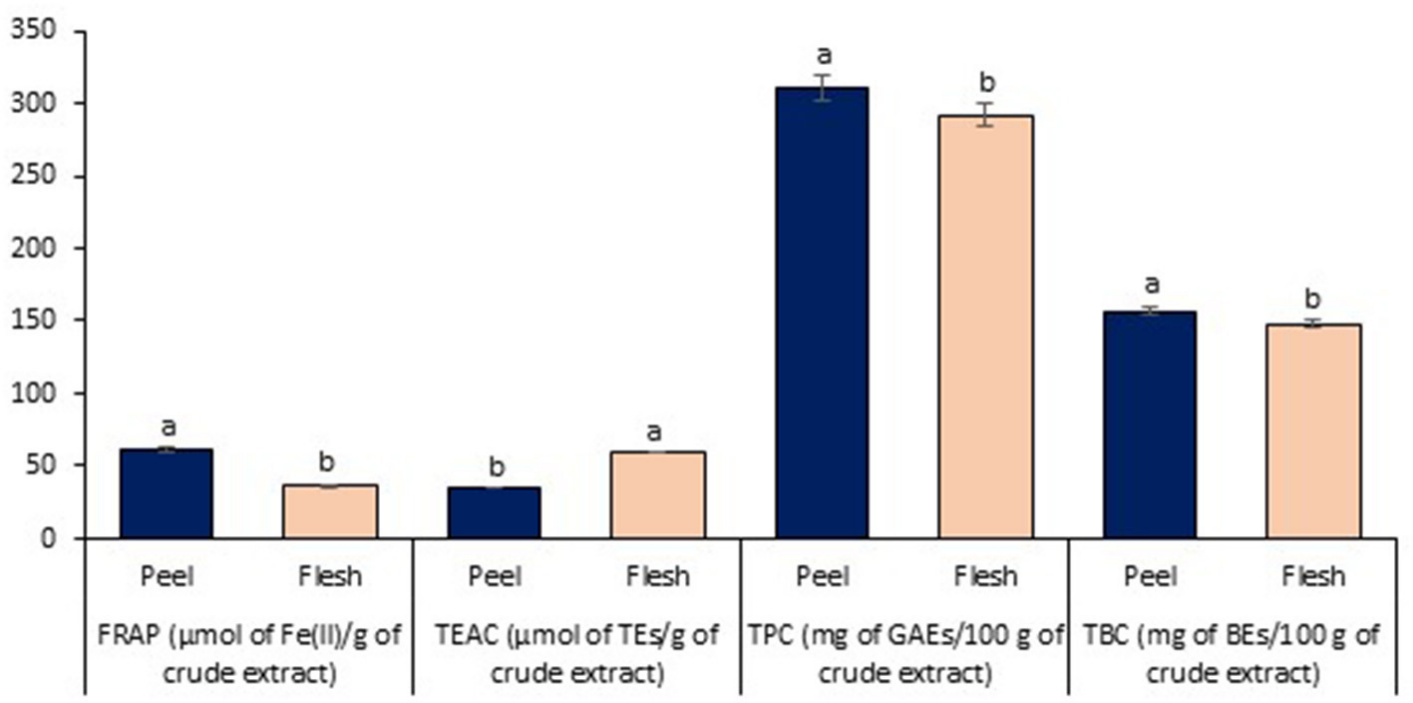
Antioxidant properties assessment of peel and flesh extract of red-purple pitaya obtained from the optimized LBEPS. Values are mean ± SD of triplicate readings. Different letter(s) represent a significant difference (*p* < 0.05) using Tukey's test within FRAP, TEAC, TPC, and TBC.

Lastly, the antioxidant properties on peel and flesh extract of red-purple pitaya (ethanol-rich top phase) were assessed in order to examine their antioxidant capability and the presence of antioxidant compound. These extracts were obtained from the optimized LBEPS. Particularly, FRAP and TEAC are used to measure the antioxidant capability, while TPC and TBC determined the quantity of antioxidant compound (Dai and Mumper, 2010). In the FRAP assessment, the peel extract (61.767 ± 1.460 μmol of Fe(II)/g of crude extract) showed a significant higher FRAP value compared to that of the flesh extract (35.916 ± 0.489 μmol of Fe(II)/g of crude extract) (standard equation: A_593_ = 0.0006([FeSO_4_·7H_2_O]) + 0.0708 (*R*^2^ = 0.9984); A_593_: absorbance value at 593 nm & [FeSO_4_·7H_2_O]: concentration of FeSO_4_·7H_2_O). On the other hand, the peel extract (35.460 ± 0.443 μmol of TEs/g of crude extract) showed a significant lower TEAC value compared to that of the flesh extract (59.606 ± 0.857 μmol of TEs/g of crude extract) [standard equation: Percentage of scavenging (%) = 0.0966([Trolox]) + 2.8333 (*R*^2^ = 0.9946)]. Other than that, in the TPC and TBC assessment, the peel extract showed the significant higher values of TPC (310.741 ± 8.486 mg of GAEs/100 g of crude extract) and TBC (156.877 ± 2.655 mg of BEs/100 g of crude extract) compared to that of the flesh extract (TPC: 292.019 ± 7.517 mg of GAEs/100 g of crude extract, TBC: 147.840 ± 3.038 mg of BEs/100 g of crude extract); standard equation for TPC: A_725_ = 0.0036([gallic acid]) + 0.0086 (*R*^2^ = 0.9995) (Figure 7). Our results concluded that electricity treatment did not reduce the antioxidant properties from the red-purple pitaya, but in fact it enhanced them as the antioxidant values obtained in this study are much higher compared to the previously reported studies (Fu et al., 2011; Ramli et al., 2014). Moreover, our Pearson's correlation study conveyed that the FRAP with TEAC, TPC, and TBC were correlated among them with a significant strong positive relationship. TEAC showed a non-significant strong negative with TPC (*p* > 0.05), whereas a significant strong negative relationship between TEAC and TBC was noted. TPC and TBC demonstrated a non-significant strong positive relationship (Table 3).

**Table 3:** Correlation study among the antioxidant properties, given correlation coefficient (*r*).

	**TEAC**	**TPC**	**TBC**
FRAP	−0.997^**^	0.853^*^	0.899^*^
TEAC	–	−0.811	−0.902^*^
TPC	–	–	0.788

** *Correlation is significant at p < 0.01 (2-tailed)*.

* *Correlation is significant at p < 0.05 (2-tailed)*.

## Conclusions

This study concludes that a satisfactory betacyanins extraction from the peel and flesh of red-purple pitaya was successfully achieved with the utilization of the optimized LBEPS. In the system, electricity treatment greatly improves extraction of biomolecules like betacyanins. In addition, the peel and flesh extract showed different variations of red color, and they are proved to demonstrate appreciable antioxidant properties. Overall, this article introduces a new, easy and effective green bioseparation technology for the biomolecules separation which could be applied in the downstream bioprocessing industries. For instance, a pilot-scale LBEPS on the biomolecules separation could be investigated, owing to its high potential to serve as an effective bioseparation technology.

## Author Contributions

HL performed the experiment and data analysis as well as wrote the manuscript. Y-KC and CO revised the manuscript. CL, AJ, and PS conceived and designed the experiment.

## Correction note

This article has been corrected with minor changes. These changes do not impact the scientific content of the article.

## Conflict of Interest Statement

The author PS had previously collaborated with the reviewer NTDP.

The remaining authors declare that the research was conducted in the absence of any commercial or financial relationships that could be construed as a potential conflict of interest.
