# Expression of concern: Liquid biphasic electric partitioning system as a novel integration process for betacyanins extraction from red-purple pitaya and antioxidant properties assessment

**DOI:** 10.3389/fchem.2024.1450323

**Published:** 2024-07-08

**Authors:** Frontiers Editorial Office

In the published article, the conflict of interest lacked necessary details regarding a previous collaboration between author Pau Loke Show & reviewer Nguyen Thi Dong Phuong. An investigation by the Research Integrity auditing team found that this conflict of interest did not unduly impact the peer review process or scientific validity of the article.

As a result, a Correction has been published, and the Expression of Concern is considered resolved. This notice remains published to retain a transparent record.

