# Correction: Liquid biphasic electric partitioning system as a novel integration process for betacyanins extraction from red-purple pitaya and antioxidant properties assessment

**DOI:** 10.3389/fchem.2025.1702149

**Published:** 2025-09-22

**Authors:** Frontiers Editorial Office

**Affiliations:** Frontiers Editorial Office, Frontiers Media SA, Switzerland

In the published article, the conflict of interest lacked necessary details regarding a previous collaboration between author Pau Loke Show and reviewer Nguyen Thi Dong Phuong. An investigation by the Research Integrity auditing team found that this conflict of interest did not unduly impact the peer review process or scientific validity of the article.

The correct conflict of interest statement appears below

“The author PS had previously collaborated with the reviewer NTDP.

The remaining authors declare that the research was conducted in the absence of any commercial or financial relationships that could be construed as a potential conflict of interest.”

The original article has been updated.

